# Supplementary material for: Donor Genetic Predisposition to High Interleukin-10 Production Appears Protective against Acute Graft-Versus-Host Disease
Source: Int J Mol Sci. 2022 Dec 14;23(24):15888. doi: 10.3390/ijms232415888 (PMC9779827; doi:10.3390/ijms232415888)
Supplement: Supplementary file 1 [file ijms-23-15888-s001.zip › ijms-2070963-supplementary.pdf]

Supplemental Figure

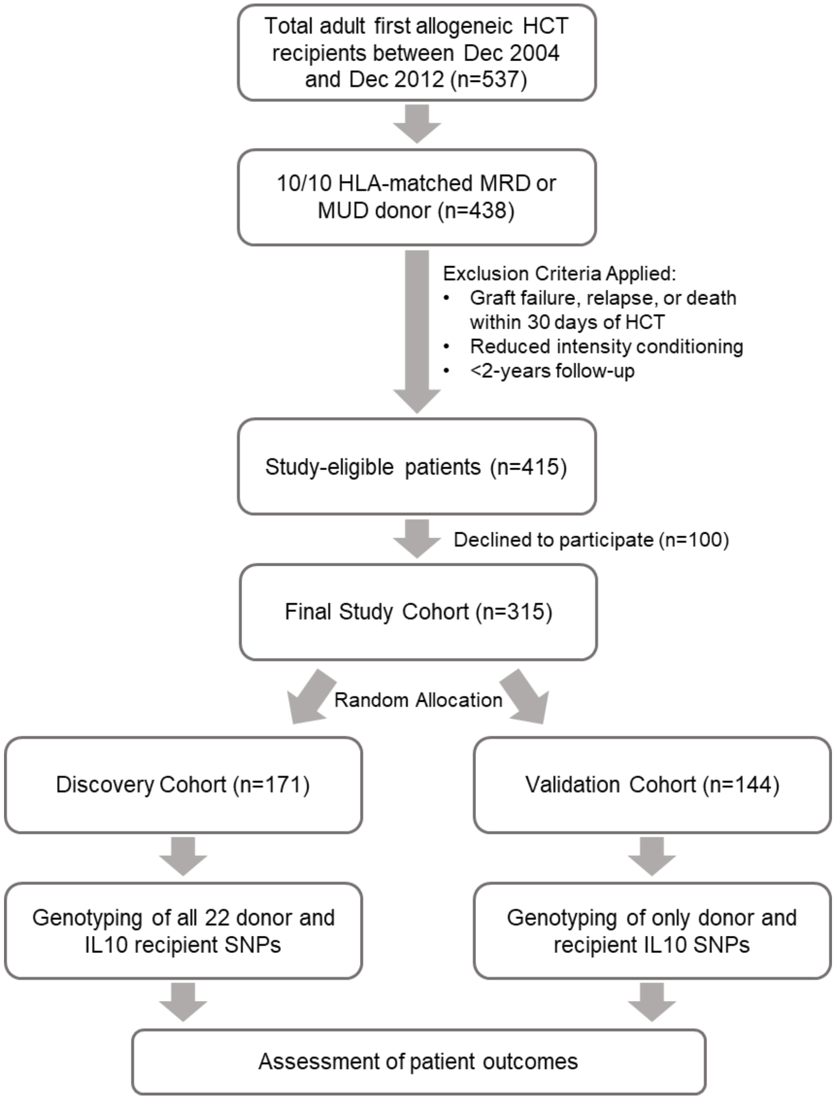

Supplemental Figure S1. Flow chart representing the cohort selection criteria and study design

Supplemental Tables

Table S1. Prevalence of single nucleotide polymorphisms among study cohort

Single Nucletide Polymorphism (SNP)	Prevalence*		
IL10-1082 (rs1800896)	AA (19.7%)	AG (62.9%)	GG (17.5%)
IL10-819 (rs1800871)	TT (6.8%)	CT (39.2%)	CC (54.1%)
IL10-592 (rs1800872)	CC (7.4%)	CA (38.2%)	AA (54.4%)
IL1R+1970 (rs2234650)	CC (41.9%)	CT (10.4%)	TT (47.7%)
IL1a-889 (rs1800587)	CC (56.1%)	CT (7.6%)	TT (43.4%)
IL1b-511 (rs16944)	CC (44.4%)	CT (12.1%)	TT (43.4%)
IL1b+3962 (rs1143634)	CC (60.6%)	CT (8.6%)	TT (30.8%)
IL1RA-11100 (rs315952)	CC (6.6%)	CT (47.5%)	TT (46.0%)
IL4Ra+1902 (rs1801275)	AA (66.0%)	AG (5.6%)	GG (28.4%)
IL12-1188 (rs3212227)	AA (61.6%)	CA (7.1%)	CC (31.3%)
IFNγ+874 (rs2430561)	AA (35.9%)	AT (19.7%)	TT (44.4%)
TGFb codon 10 (rs1982073)	CC (19.3%)	CT (28.4%)	CC (52.3%)
TGFb codon 25 (rs1800471)	CC (1.5%)	CG (85.8%)	GG (12.7%)
TNFa-308 (rs1800629)	GG (56.6%)	GA (2.0%)	AA (41.4%)
TNFa-238 (rs361525)	GG (87.9%)	GA (0.5%)	AA (11.6%)
IL2-330 (rs2069762)	TT (51.0%)	TG (7.6%)	GG (41.4%)
IL2+166 (rs2069763)	GG (14.6%)	GT (43.9%)	TT (41.4%)
IL4-1098 (rs2243248)	GG (1.5%)	GT (78.3%)	TT (20.2%)
IL4-590 (rs2243250)	CC (65.7%)	CT (6.1%)	TT (28.3%)
IL4-33 (rs2070874)	CC (70.9%)	CT (8.7%)	TT (20.4%)
IL6-174 (rs1800795)	CC (18.7%)	CG (33.8%)	GG (47.5%)
IL6-565 (rs1800797)	AA (14.1%)	AG (33.8%)	GG (52.0%)

*Prevalence for IL10 SNPs determined in total study cohort; prevalence for other SNPs determined only in discovery cohort.

Table S2. Competing risks for regression analyses

Outcome	Competing Risk
Grade II-IV acute GVHD	Graft failure, relapse, second malignancy, moderate-severe cGVHD, or death
Moderate-severe chronic GVHD	
NST	Graft failure, relapse, second malignancy, or death
Clinically significant GVHD*	Graft failure, relapse, second malignancy, or death
Relapse	Graft failure, second malignancy, or non-relapse death
Non-relapse mortality	Relapse

*Clinically significant GVHD is defined as grade II-IV acute GVHD or moderate-severe cGVHD

Table S3. Covariates for regression analyses

Variable*	Gr II-IV aGVHD	Mod-Sev cGVHD	sGVHD	Relapse	OS
Patient Age (<45 vs. ≥45 years)	0.180	0.060	0.188	0.577	**0.038**
Donor Age (<45 vs. ≥45 years)	0.423	0.226	0.934	0.841	0.125
Donor Type (MRD vs. MUD)	0.089	0.443	0.177	0.455	0.561
D/R Sex (M/M vs. other)	0.086	0.121	0.067	0.846	0.784
Graft Source (PBSC vs. BM)	0.651	0.711	0.665	0.252	0.847
Disease Risk (low vs. high)	0.188	0.126	0.095	**0.049**	0.194
Conditioning Regimen (No TBI vs. TBI)	**0.049**	**<0.001**	**0.002**	0.226	0.490
D/R CMV Serostatus					
D-R- vs. other	0.777	0.600	0.591	0.524	0.280
D-R+ vs. other	0.513	0.536	0.330	0.612	0.955
D+R- vs. other	0.488	0.652	0.993	0.336	**0.001**
D+R+ vs. other	0.679	0.648	0.782	0.984	**0.047**
D/R EBV Serostatus					
D-R- vs. other	0.187	0.812	0.085	#	1.00
D-R+ vs. other	0.155	0.677	0.178	0.910	0.337
D+R- vs. other	0.305	0.556	0.321	0.901	0.710
D+R+ vs. other	0.878	0.951	0.970	0.679	0.353

*Covariates that were statistically significant (two-tailed p<0.05) were included in multivariate regression analyses.

#Insufficient sample size in covariate for statistical comparison.

Abbreviations: aGVHD = acute graft-versus-host disease (GVHD); cGVHD = chronic GVHD; sGVHD = clinically significant GVHD; OS = overall survival; NRM = non-relapse mortality; MRD = 8/8 HLA matched related donor; MUD = 8/8 HLA matched unrelated donor; D/R = donor-recipient; PBSC = peripheral blood stem cells; BM = bone marrow; Flu = fludarabine; Bu = busulfan; TBI = total body irradiation; CMV = cytomegalovirus; EBV = Epstein-Barr virus

Table S4. Association of donor single nucleotide polymorphisms with GVHD outcomes

Single Nucleotide Polymorphism (SNP)*	Grade II-IV aGVHD			Moderate-Severe cGVHD			sGVHD		
	p-value	SHR	95% CI	p-value	SHR	95% CI	p-value	SHR	95% CI
IL10-1082 (rs1800896)									
Discovery Cohort	**0.023**	0.24	0.07-0.82	0.293	0.51	0.14-1.81	**0.012**	0.26	0.09-0.74
Validation Cohort	**0.037**	0.22	0.05-0.91	0.51	0.72	0.26-1.92	**0.026**	0.34	0.13-0.88
Total Patients	**0.005**	0.25	0.09-0.66	0.148	0.55	0.24-1.23	**0.001**	0.33	0.17-0.65
MRD Patients	0.102	0.41	0.14-1.16	0.52	0.69	0.21-2.15	0.09	0.47	0.20-1.12
MUD Patients	**0.008**	0.15	0.03-0.61	0.207	0.47	0.14-1.51	**0.021**	0.27	0.09-0.82
IL10-819 (rs1800871)									
Discovery Cohort	0.065	0.08	0.005-1.16	0.266	0.12	0.05-0.24	0.156	0.37	0.09-1.44
Validation Cohort	0.499	0.66	0.19-2.19	0.484	1.56	0.45-5.47	0.94	1.03	0.39-2.71
Total Patients	0.088	0.49	0.21-1.11	0.287	1.84	0.59-5.72	0.319	0.69	0.34-1.41
MRD Patients	0.182	0.42	0.12-1.48	0.251	2.4	0.53-10.77	0.51	0.73	0.29-1.85
MUD Patients	0.304	0.51	0.14-1.85	0.763	1.29	0.23-7.05	0.429	0.66	0.23-1.86
IL10-592 (rs1800872)									
Discovery Cohort	0.093	0.31	0.08-1.22	0.181	0.13	0.05-0.24	0.295	0.52	0.16-1.76
Validation Cohort	0.48	0.61	0.15-2.40	0.501	1.51	0.45-4.93	0.964	1.02	0.40-2.58
Total Patients	0.068	0.45	0.19-1.06	0.245	1.92	0.63-5.79	0.389	0.73	0.37-1.47
MRD Patients	0.12	0.37	0.11-1.29	0.217	2.54	0.57-11.22	0.611	0.79	0.32-1.96

MUD Patients	0.684	0.74	0.17-3.17	0.701	1.37	0.27-6.99	0.472	0.67	0.23-1.95
IL1R+1970 (rs2234650)	0.155	1.75	0.81-3.81	0.544	0.78	0.35-1.75	0.077	1.73	0.94-3.17
IL1a-889 (rs1800587)	0.121	0.21	0.03-1.51	0.845	0.91	0.34-2.43	0.212	0.58	0.24-1.36
IL1b-511 (rs16944)	0.474	0.71	0.27-1.82	0.118	0.47	0.19-1.21	0.114	0.51	0.22-1.17
IL1b+3962 (rs1143634)	0.926	0.95	0.34-2.63	0.321	1.52	0.66-3.49	0.359	1.35	0.71-2.59
IL1RA-11100 (rs315952)	0.48	0.62	0.16-2.31	0.547	1.43	0.44-4.62	0.956	0.97	0.35-2.63
IL4Ra+1902 (rs1801275)	0.165	0.26	0.04-1.71	0.943	0.96	0.34-2.68	0.301	0.62	0.24-1.54
IL12-1188 (rs3212227)	0.238	0.29	0.04-2.24	0.605	0.7	0.18-2.68	0.403	0.64	0.22-1.82
IFNγ+874 (rs2430561)	0.583	1.24	0.57-2.70	0.899	1.04	0.50-2.20	0.288	1.37	0.76-2.47
TGFb codon 10 (rs1982073)	0.295	1.69	0.63-4.52	0.512	0.79	0.39-1.59	0.789	1.09	0.57-2.08
TGFb codon 25 (rs1800471)	0.09	0.23	0.09-4.41	0.842	0.84	0.15-4.53	0.643	1.47	0.28-7.69
TNFa-308 (rs1800629)	0.841	1.25	0.13-11.37	0.792	1.13	0.46-2.80	0.245	2.07	0.61-7.11
TNFa-238 (rs361525)	0.124	2.41	0.99-6.06	0.191	2.23	1.12-4.12	0.122	0.23	0.08-2.21
IL2-330 (rs2069762)	0.234	1.79	0.68-4.71	0.21	1.78	0.72-4.43	0.101	1.96	0.87-4.39
IL2+166 (rs2069763)	0.901	0.93	0.32-2.69	0.795	1.11	0.49-2.48	0.788	1.1	0.53-2.26
IL4-1098 (rs2243248)	0.211	0.49	0.14-1.7	0.591	1.86	0.19-17.91	0.425	2.24	0.31-16.47
IL4-590 (rs2243250)	0.498	1.41	0.52-3.88	0.112	2.32	1.25-4.31	0.191	2.26	1.21-4.23
IL4-33 (rs2070874)	0.217	1.71	0.72-4.02	0.08	1.75	0.93-3.29	0.078	1.66	0.94-2.95

IL6-174 (rs1800795)	**0.015**	0.52	0.26-1.05	0.824	0.92	0.45-1.87	0.088	0.59	0.32-1.08
IL6-565 (rs1800797)	0.14	0.48	0.18-1.26	0.69	1.18	0.52-2.67	0.459	0.76	0.37-1.55

Abbreviations: aGVHD = acute graft-versus-host disease (GVHD); cGVHD = chronic GVHD; sGVHD = clinically significant GVHD

Table S5. Association of recipient IL10 single nucleotide polymorphisms with GVHD outcome

Single Nucletide Polymorphism (SNP)	Grade II-IV aGVHD			Moderate-Severe cGVHD			sGVHD		
	p-value	SHR	95% CI	p-value	SHR	95% CI	p-value	SHR	95% CI
IL10-1082 (rs1800896)									
Total Patients	0.379	0.73	0.37-1.45	0.418	0.77	0.41-1.44	0.186	0.71	0.42-1.18
MRD Patients	0.994	0.99	0.36-2.73	0.937	0.96	0.38-2.40	0.967	0.98	0.45-2.12
MUD Patients	0.273	0.58	0.22-1.52	0.304	0.62	0.25-1.53	0.114	0.56	0.28-1.14
IL10-819 (rs1800871)									
Total Patients	0.653	1.23	0.50-3.01	0.243	1.73	0.69-4.35	0.419	1.34	0.65-2.76
MRD Patients	0.423	1.74	0.45-6.81	0.221	2.51	0.57-10.95	0.209	2.11	0.65-6.83
MUD Patients	0.874	0.9	0.25-3.15	0.739	1.19	0.42-3.36	0.794	0.88	0.35-2.18
IL10-592 (rs1800872)									
Total Patients	0.45	1.22	0.50-3.01	0.243	1.73	0.69-4.35	0.419	1.34	0.66-2.77
MRD Patients	0.423	1.74	0.45-6.81	0.221	2.51	0.57-10.95	0.209	2.11	0.65-6.83
MUD Patients	0.874	0.91	0.25-3.15	0.739	1.19	0.42-3.36	0.794	0.88	0.35-2.18

Abbreviations: aGVHD = acute graft-versus-host disease (GVHD); cGVHD = chronic GVHD; sGVHD = clinically significant GVHD

DNA Extraction and Genotyping

The panel of SNPs tested include tumor necrosis factor-α (-308G/A and -238G/A), interferon-γ (+874A/T), interleukin (IL)-6 (-174G/C and nt565G/A), IL-1α (-889T/C), IL-1β (-511C/T, +3962T/C), IL-1 receptor (IL-1R) (pst1 -1970C/T), IL-1 receptor agonist (mspal -11100T/C), IL-2 (-330T/G and +166G/T), IL-4 (-1098T/G, -590T/C and -33T/C), IL-4Rα (+1902G/A), IL-12 (-1188C/A), IL-10 (-1082G/A, -819C/T, and -592C/A), and transforming growth factor-β (+869C/T and +915G/C). The presence or absence of PCR products was visualized by 2% agarose gel electrophoresis. After electrophoresis, the gel was placed on an ultraviolet trans-illuminator, and photography for interpretation and documentation was performed. Each of the primer mixes contained a control primer pair that amplified either a part of the-globin gene (89bp) or a part of the C-reactive protein gene (440bp). In cases where there was insufficient DNA in specimens to conduct SSP-PCR, whole genome amplification (WGA) was conducted[1] according to manufacturers protocol (Qiagen REPLI-g UltraFast Mini Kit).

In short, the PCR was done using following primers: forward; 5’-ATCCAAGACAACACTACTAA-3’ and reverse; 5’-TAAATATCCTCAAAGTTCC-3’. These primers yielded an amplicon of 587bp in size (-1115 to -528) containing the above three polymorphisms of IL-10 gene. The amplification was performed in a 20μL medium containing 1μL (100ng/μL) of template DNA, Taq DNA polymerase 2.5 units, PCR buffer 2μL (10X), forward and reverse primers 1μL each (10pM), MgCl2 (50mM) 2μL, dNTPs 1μL (5mmol/L) and nuclease free water 11μL. The PCR cycle conditions consisted of an initial denaturation step at 95°C for 5 min, followed by 30 cycles of 30s at 95°C; 30s at 58.7°C; 60s at 72°C; a final elongation at

72°C for 5 min and then storage at 40°C. After confirming the final products by electrophoresis on agarose gels (2%), the PCR products were re-sequenced using the core facility at University of Calgary, Canada. Nucleotide polymorphisms at positions -1082, -819, and -592 were read directly.

Determination of *IL-10* transcripts by Nanosting assay

Total RNA was extracted from 5 million cells using the RNeasy Mini Kit from Qiagen, Germany. 100ng of total RNA was used an input material for the Nanostring Assay. Reporter probe with the fluorescence barcode at the 5` end and capture probe carrying the biotin on the 3` end were added to the reaction mix along with the sample according to manufacturer`s instructions. Hybridization of the probes with the targeted mRNA was allowed to take place at 65 C for 20 hours. A total of 12 samples were run in batches making sure to include biological replicates in each batch. Excess probes were washed using magnetic beads and the purified target/probe complexes were immobilized on the cartridge using the automated nCounter Prep Station. The cartridge was read in the nCounter digital Analyzer. Gene expression raw counts were normalized using the nSolver software from Nanostring Technologies. The assay contains internal reference positive and negative controls and housekeeping gene transcripts (e.g., GBPD, GAPDH, OAZ1, POLR1B, and TUBB) that were used as reference to normalize the raw IL10mRNA counts from the samples. Positive control normalization to correct for the assay related variations was done using the geometric mean of the Internal positive control counts for all samples. Reference gene normalization was conducted to correct for variations in the sample input between assays using the

geometric mean of 7 housekeeping gene counts included in the codeset. Background threshold (negative control normalization) was calculated using the geometric mean of the negative control counts included in the codeset and was subtracted from the gene counts.

Enumeration of Immune Cell Subsets by Multicolor Flow-Cytometry

Heparinized blood (200uL) was pipetted into 12 x 75 mm polystyrene tubes and washed with phosphate buffered saline (PBS). Monoclonal antibodies labelled with fluorochromes were added to the cell pellet and incubated at 15 minutes at room temperature. Antibodies used were anti-human CD45RA-PE, anti-human CD3-ECD, anti-human CD8-PC5, anti-human CD4-PE-Cy7. All antibodies were obtained from Beckman Coulter/Immunotech (Miami, FL). Erythrocytes were lysed using ammonium chloride lysing solution and washed with PBS. Cells were resuspended in 0.5mL of 0.1% formaldehyde in PBS. Flow cytometric analysis was performed using FC500 flow cytometer (Beckman Coulter, Hialeah, FL). Lymphocytes were gated in the SSC vs FSC plot followed by ECD positive $CD3^+$ T cells. $CD4^+$ and $CD8^+$ gates were made in the population coming from parent $CD3^+$ cells. Percentage $CD3^+$ T cells out of the parent lymphocyte population were compared between the GG and AA genotypes. Percentage $CD4^+$ and $CD8^+$ cells out of the parent CD3+ T cells population were compared between GG and AA genotypes.

Donor Chimerism Assessment

DNA was extracted from peripheral blood cells from the donor and recipient pre-transplant using the QIAamp DNA Micro-Kit (QIAGEN Inc., Hilden, Germany) for the initial determination of donor and recipient genotype. Peripheral blood samples were obtained from recipients at one-month post-transplant and sorted for $CD3^{+}$ T-cells and $CD13^{+}CD33^{+}$ myeloid cells using the BD FACS Aria cell sorter. Chimerism assessment was conducted using the Identifiler STR (short tandem repeat) panel kit (AmpFISTR® Identifiler® Plus PCR Amplification Kit) from Applied Biosystems, ThermoFisher Scientific. The kit included 4-color fluorescence-tagged primers against 16 STR markers on 14 chromosomes. The targets in donor and recipient DNA were amplified in multiplex PCR conducted according to manufacturer's protocol. Amplicons were size-fractioned by capillary electrophoresis on the ABI-3130 Genetic Analyzer and chimerism was determined by quantifying the relative proportion of uniquely identified donor- and recipient-specific alleles using GeneMapper software.

References:

1. Khan F, Liacini A, Arora E, et al. Assessment of fidelity and utility of the whole-genome amplification for the clinical tests offered in a histocompatibility and immunogenetics laboratory. *Tissue antigens*. May 2012;79(5):372-9. doi:10.1111/j.1399-0039.2012.01857.x
